# Some Interesting Autopsies

**Published:** 1883-11

**Authors:** Frederick Peterson


### Some Interesting Autopsies.

By Frederick Peterson, M. D.

(*Continued from September and October numbers of the Journal.*)

### NO. 28—PNEUMONIA. CHRONIC MENINGITIS. HEMORRHAGE IN PONS VAROLII.

State Insane Asylum, March 27—David Davis, æt. 67; dead sixteen hours; body well nourished; to left of larynx, wound 4½ cm. in diameter in process of healing (suicidal attempt); bruises upon left knee and elbow joints.

*Head:* Dura mater normal; pia mater thickened; puncta vasculosa very marked; ependyma of ventricles thickened; in left side pons varolii a small spot 6 mm. in diameter, full of minute capillary ecchymoses, giving to this spot a dark red tinge altogether different from the white substance of the pons. Arteries at base of brain markedly degenerated (endarteritis deformans).

*Chest:* Right pleural cavity contained two litres of serum full of flocculent fibrin; pleura covered, on lower part of lung, with fibrin; emphysema of lungs (senile atrophy the cause); left pleura and cavity normal; middle and lowest lobes of right lung and lower lobe of left lung in stage of red hepatization (croupous pneumonia); upper lobe of left lung acutely congested and œdematous. Marks of old pericarditis; in pericardium some sixty-four grammes of clear serum; valves normal; beginning endarteritis deformans in aorta and at bases of lunettes of aortic valve; myocardium fatty.

*Abdomen:* Spleen soft and friable; contained an infarct 4 cm. in diameter. Liver a little enlarged and somewhat fatty. Granular atrophy of both kidneys. Bladder and ileum normal. Stomach distended; exceedingly thin-walled; there seemed to be no destruction of mucous membrane, yet could see through the walls, so thin were they; tore easily; seemed as if muscular coat were absent; great congestion of the vessels of mucosa, with numerous extensive hemorrhages into stomach tissues (took arsenic some time before for suicidal purposes); chronic gastric catarrh.

*Diagnosis:* Pneumonia cause of death. Insanity from chronic meningitis. Chronic interstitial nephritis. Chronic gastritis. Apoplexy in pons varolii.

*Remarks:* The extravasation in left side of pons varolii caused dilatation of right pupil and convulsions.

NO. 30—ACUTE MILIARY TUBERCULOSIS OF LUNGS, LIVER, KIDNEYS AND MENINGES.

Buffalo General Hospital, April 3—P. Balderspergen, male, æt. 45; dead fourteen hours. Body in fairly nourished condition; rigor mortis marked; physique remarkably fine.

*Head:* Pia and arachnoid inflamed and contained numerous minute tubercles.

*Chest:* Right and left lungs completely full everywhere of miliary tubercles; in right apex red hepatization from pneumonia; strong pleuritic adhesions on both sides. Heart in every way normal; beginning atheroma in aorta; signs of old pericarditis.

*Abdomen:* Spleen soft. Liver of normal size and full of minute tubercles scarcely visible to the naked eye. Both kidneys of normal size and containing numerous tubercles. Bladder distended with urine. Ileum normal. Pyloric end of stomach somewhat inflamed with numerous ecchymoses. Some tubercles on peritoneum.

*Diagnosis:* Acute miliary tuberculosis of lungs, liver, kidneys and meninges.

*Remarks:* Shortly before death, patient was attacked with convulsions. One pupil was dilated. He died comatose.

### NO. 40—CHRONIC GENERAL TUBERCULOSIS OF LUNGS, SPLEEN, KIDNEYS, RENAL PELVIS, PROSTATE GLAND, PERITONEUM AND INTESTINES.

Buffalo General Hospital, May 14—Kleinholz, æt. 53, male; dead twenty hours. Body long and emaciated; rigor mortis still present; small tumor 2 cm. in diameter over sternum, subcutaneous, full of cheesy matter (atheroma).

Head not examined.

*Chest:* Agonia clots in right heart; some atheromatous disease of aorta; myocardium somewhat fatty. Both lungs masses of tubercles and vomicæ, containing pus; everywhere strong pleuritic adhesions.

*Abdomen:* Spleen atrophied, soft, and contained two or three tubercles; capsule much thickened and partly cartilaginous. Liver small and fatty. Left kidney full of miliary tubercles; tubercular pyelitis, pelvis being full of pus and tubercles standing out from mucosa like granulations; pelvis also dilated and the ureter much larger than normal. Right kidney: pelvis normal; ureter smaller than normal, like a fine cord, but pervious; great amount of fat about pelvis; kidney structure normal. Prostate gland double its normal size and contained a large cheezy tubercle size of a hazel nut. Chronic cystitis. Testicles and epididymes normal. Mucosa of stomach covered with innumerable hemorrhagic erosions. Numerous ulcers in ileum, with tubercles in their borders. Upon the mesentery of bowels, a number of tubercles of small size.

*Diagnosis:* Chronic general tuberculosis.

*Remarks:* Tubercles in the prostate are very rare.

NO. 38—ACUTE TUBERCULAR MENINGITIS.

Buffalo General Hospital, May 9—A. Adel, æt. 21, male; dead twenty-one hours. Body small and emaciated; left chest sunken in; circumcised.

*Head:* Calvarium very thin and brachycephalic; great congestion of diploic veins, also of sinuses of dura mater and veins of pia mater. Dura mater thin and translucent. Pia mater and arachnoid thickened, opaque, and with here and there minute white points just visible to the naked eye, which, under the microscope, proved to be tubercles. At base of brain more than usual quantity of serum. Ventricles widened and full of red-tinged serum; ependyma soft. Brain substance itself normal.

*Chest:* Right pleura not adherent; left everywhere strongly bound to thorax. Right lung contained eight or ten small tubercles in apex, but otherwise normal. Left lung atalectatic and red hepatized. In axillary region the fourth and fifth ribs were broken, each in two places, about 10 cm. apart, the two fragments thus made being dislocated outward; some union had taken place; one of the inward projecting portions of rib, the fifth, was necrosed, and about it was an encapsuled inter-pleural cavity some 5 cm. in diameter, containing no fluid. Heart small.

*Abdomen:* Spleen and liver small, but normal. Kidneys normal. Bladder distended with urine. Stomach and ileum normal.

*Diagnosis:* Acute tubercular meningitis, with hydrocephalus internus acutus. Further croupous pneumonia of left lung, probably as a result of a comparatively recent fracture of ribs and pleuritic inflammation and suppuration.

*Remarks:* He entered the hospital but a few days before death, and his symptoms were chiefly those of the meningitis, with rapidly supervening coma. The fracture or pneumonia were therefore naturally unsuspected, and no history of a fall could be obtained even from his friends. Until within a few weeks he had been at work in a dry goods store as clerk.

### NO. 43—SUBACUTE GENERAL TUBERCULOSIS.

Buffalo General Hospital, May 17—Reitz, æt. 62, male; dead nine hours. Body well formed, large; rigor mortis marked.

Head not examined.

*Chest:* In right pleural cavity three litres of clear serum; in left about one litre and a few adhesions; both lungs full of miliary tubercles; lower lobes consolidated. Pericardium thickened and adherent; heart normal.

*Abdomen:* Left kidney enlarged, hyperæmic, and contained several tubercles; right kidney much enlarged, hyperæmic, and full of tubercles. Liver enlarged and contained numerous tubercles, also very fatty. Spleen enlarged and contained several tubercles. In stomach some catarrhal inflammation. Ileum and bladder normal.

*Diagnosis:* Subacute general tuberculosis.

### NO. 44—ACUTE TUBERCULOSIS OF MENINGES, LUNGS AND KIDNEYS, IN A SEVEN MONTHS' PREGNANT WOMAN. DILATATION OF RIGHT URETER ABOVE BRIM OF PELVIS FROM PRESSURE OF CHILD. INTERESTING ANATOMICAL POINTS.

Buffalo General Hospital, May 18—Margaret Z., æt. 30, emigrant; dead fifteen hours Body well nourished; abdomen enlarged by seven months' child; rigor mortis marked.

*Head:* Dura mater normal. Numerous small tubercles in basilar meninges. Brain substance normal.

*Chest:* Strong adhesions of left pleura; left lung œdematous, full of miliary tubercles and consolidated in upper lobe. Strong adhesions also on right side; right lung also full of miliary tubercles, and œdematous. Heart normal.

*Abdomen:* Peritonitis over diaphragm, liver and spleen. Spleen contained numerous tubercles. Liver enlarged, soft and pale (cloudy swelling). In left kidney a few miliary tubercles and fatty degeneration; left ureter normal. Ureter of right kidney dilated just above brim of pelvis to four times its normal size, due to direct pressure of the gravid uterus, which leaned way over to right side and did not press at all upon left ureter; right kidney enlarged, fatty, and contained some tubercles (first stage of parenchymatous nephritis). There were no tubercles to be found anywhere in the child, although careful search was made. This case was anatomically interesting. The uterus was very thin, not more than 1–2 mm. thick. The internal os was not open and neck not dilated. In the right ovary was the corpus luteum of the child, with its wrinkled, stellate border. The ureters could be traced down through the base of the broad ligament to their outlet into the bladder, an interesting point in pregnancy.

*Diagnosis:* Direct cause of death asthenia, from tuberculosis of meninges, lungs and kidneys.

### NO. 59—GENERAL TUBERCULOSIS OF PERITONEUM, LIVER, KIDNEYS, MENINGES, LUNGS AND MEDULLA OBLONGATA.

Buffalo General Hospital, August 2—D. Bisantz, male, æt. 33; dead twenty-two hours. Body emaciated; teeth good; no enlarged glands; no sign of cicatrix on penis; a few copper-colored patches size of pea on both legs.

*Head:* Calvarium thin; dura mater on left side normal; on right side under surface a slight old pachymeningitis and numerous excrescences, soft and yellow, from size of millet seed to four lines in diameter; on pia mater, over right hemisphere, also numerous of these yellowish excrescences, some protuding externally, others going deep into the white substance of the brain (seven of these were the size of hazel nuts, with soft, mushy, yellow interior; brain softened about them; all united by thin pedicles to pia from which they sprung). Both lateral ventricles enlarged, ependyma softened, contained blood-tinged serum. Pia and arachnoid everywhere thickened, but contained no unusual amount of serum. Pons normal. Very minute miliary tubercles on pia in fossæ sylvii. A hard, white tumor, size of hazel nut, in right side of medulla oblongata, close to pons varolii, including anterior pyramid and olivary body of right side. Cerebellum normal.

*Chest:* Depressed on right side externally. In right pleural cavity two litres of serum full of fibrin; lung completely atalectatic and pushed up to apex of chest; pleura covered with fibrin. In left lung numerous cheesy tubercles from size of pin's head to that of pea and larger; no adhesions. Chronic bronchitis. Pericardium so adhered by old inflammation that it was impossible to remove it from heart; heart small; muscle normal; valves all normal; aorta dilated and covered everywhere on intima with smooth protuberances; just in front of aorta ascendens, at junction with the arch, a sacculated aneurism some 5 cm. in diameter externally, with thick stratified walls, and would hold about 12 ccm. of blood; the opening into this was 1½ cm. wide.

*Abdomen:* Spleen enlarged and soft. The peritoneum, both visceral and parietal, everywhere covered with myriads of millet-seed growths; the omentum a perfect mass of these. Liver full of miliary tubercles. Chronic catarrh of stomach. Both kidneys enlarged, cyanotic and hard, each containing several small tubercles. Bladder and ileum normal.

*Diagnosis:* General tuberculosis of peritoneum, liver, kidneys, lungs, meninges and medulla ablongata. Farther, chronic bronchitis, gastric catarrh, pleurisy with effusion, chronic pericarditis, acute hydrocephalus internus and cyanotic induration of kidneys.

*Remarks:* This man was treated in the hospital where I was resident in 1879 for a fractured tibia and fibula. At that time he had no sign of tuberculosis. He was found this time unconscious in the street and paralyzed on the right side, and was carried to the General Hospital. No further history is given. He recovered consciousness in the hospital for a short time and seemed to improve, but finally succumbed comatose.

(*To be continued in next number.*)

